# Clinical Approach to Post-acute Sequelae After COVID-19 Infection and Vaccination

**DOI:** 10.7759/cureus.49204

**Published:** 2023-11-21

**Authors:** Nicolas Hulscher, Brian C Procter, Cade Wynn, Peter A McCullough

**Affiliations:** 1 Epidemiology, Unversity of Michigan School of Public Health, Ann Arbor, USA; 2 Family Medicine, McKinney Family Medicine, McKinney, USA; 3 Internal Medicine, Cardiology, McKinney Family Medicine, McKinney, USA; 4 Cardiology, Epidemiology, and Public Health, McCullough Foundation, Dallas, USA

**Keywords:** medicine, treatment protocol, long covid, post-acute sequelae, covid-19 vaccines, covid-19 treatment, spike glycoprotein, spike protein detoxification, sars-cov-2 spike protein, sars-cov-2

## Abstract

The spike protein of SARS-CoV-2 has been found to exhibit pathogenic characteristics and be a possible cause of post-acute sequelae after SARS-CoV-2 infection or COVID-19 vaccination. COVID-19 vaccines utilize a modified, stabilized prefusion spike protein that may share similar toxic effects with its viral counterpart. The aim of this study is to investigate possible mechanisms of harm to biological systems from SARS-CoV-2 spike protein and vaccine-encoded spike protein and to propose possible mitigation strategies. We searched PubMed, Google Scholar, and ‘grey literature’ to find studies that (1) investigated the effects of the spike protein on biological systems, (2) helped differentiate between viral and vaccine-generated spike proteins, and (3) identified possible spike protein detoxification protocols and compounds that had signals of benefit and acceptable safety profiles. We found abundant evidence that SARS-CoV-2 spike protein may cause damage in the cardiovascular, hematological, neurological, respiratory, gastrointestinal, and immunological systems. Viral and vaccine-encoded spike proteins have been shown to play a direct role in cardiovascular and thrombotic injuries from both SARS-CoV-2 and vaccination. Detection of spike protein for at least 6-15 months after vaccination and infection in those with post-acute sequelae indicates spike protein as a possible primary contributing factor to long COVID. We rationalized that these findings give support to the potential benefit of spike protein detoxification protocols in those with long-term post-infection and/or vaccine-induced complications. We propose a base spike detoxification protocol, composed of oral nattokinase, bromelain, and curcumin. This approach holds immense promise as a base of clinical care, upon which additional therapeutic agents are applied with the goal of aiding in the resolution of post-acute sequelae after SARS-CoV-2 infection and COVID-19 vaccination. Large-scale, prospective, randomized, double-blind, placebo-controlled trials are warranted in order to determine the relative risks and benefits of the base spike detoxification protocol.

## Introduction and background

Severe acute respiratory syndrome coronavirus 2 (SARS-CoV-2) is a highly contagious and pathogenic virus that emerged in late December 2019. It causes an acute respiratory disease named “coronavirus infectious disease-2019” (COVID-19). The World Health Organization (WHO) declared the outbreak of SARS-CoV-2 a pandemic on March 11, 2020 [1]. As of November 15, 2023, there have been over 700 million reported cases of COVID-19 and almost seven million deaths [2]. To combat this virus, novel gene-based vaccines were developed that utilize viral vector (AstraZeneca - ChAdOx1 nCoV-19; Johnson and Johnson - Ad26.CoV2.S) and mRNA (Moderna - mRNA 1273; Pfizer-BioNTech - BNT162b2) platforms to encode for spike protein, which elicits an immune response that generates antibodies [3]. At the time of writing, about 70% of the global population have received one or more doses of a COVID-19 vaccine [2].

The SARS-CoV-2 spike protein plays a critical role in the virus’s infection process. Spike protein emerges from the viral surface with a clove-like shape, facilitating SARS-CoV-2’s binding to host cells. This protein, which is a class I fusion transmembrane glycoprotein, is cleaved into two main sections by host proteases: the S1 and S2 subunits. The S1 subunit has a receptor-binding domain that is responsible for recognizing and binding to the host cell receptor ACE-2. Moreover, the S1 subunit is a site of drastic evolutionary pressure. Mutations of the spike protein lead to new strains that can re-infect those who have already recovered from a previous SARS-CoV-2 variant. These new variants can be more infectious than previous ones due to a higher affinity for the ACE-2 receptor. The S2 subunit is responsible for the fusion of host and viral membranes. Most of the vaccines deployed to prevent COVID-19 illness utilize a full-length spike protein stabilized in a pre-fusion state to generate immunity against SARS-CoV-2 [4].

Post-acute sequelae (long COVID) after SARS-CoV-2 infection or COVID-19 vaccination is characterized by new or persisting multi-system symptoms months to years after initial infection or vaccination [5,6]. These symptoms can range from generalized anxiety disorder and difficulty concentrating to fatigue and muscle weakness. The true incidence and prevalence of long COVID after infection are not fully known, but it has been approximated to be quite common. It is been estimated that 50% of those who were infected with SARS-CoV-2 develop long COVID, which equates to hundreds of millions of individuals [7]. Research on the prevalence of post-COVID vaccination syndrome is limited; however, a prospective cohort study found that 52.8% of individuals developed post-COVID vaccination syndrome for at least one month after primary COVID-19 vaccination. In this study, the prevalence increased to 83.9% among those who received booster doses [8]. A retrospective cross-sectional study found a post-COVID vaccination syndrome prevalence of 26.2% after COVID-19 vaccination, with higher rates seen in individuals who received Moderna vaccines compared to Pfizer vaccines [9]. These data indicate that post-acute sequelae after COVID-19 vaccination are clinically relevant; however, more research is required to find the true incidence and prevalence.

Concerns have been raised that suggest spike protein is involved in various pathologies, including post-acute sequelae after SARS-CoV-2 infection and COVID-19 vaccination [10-12]. This may be due to spike protein exhibiting harmful properties, including inflammation, endothelial tissue damage, and brain damage [10]. Moreover, spike protein has been associated with thrombotic endothelialitis, endothelial inflammation, hyperactivated platelets, and fibrinaloid microclots in those with long COVID [11]. Parry et al. found that spike protein is detrimental to many organ systems by analyzing autopsies and biopsies of spike-infected tissues [12]. If spike protein is found to be harmful to human health, it could lead to the development of new treatments that aim to reduce tissue damage caused by COVID-19. Additionally, this discovery could prompt research into ways to prevent or treat health issues resulting from COVID-19 vaccines. This is particularly relevant since COVID-19 vaccines work by using spike protein to trigger an immune response. The aim of this study is to investigate possible mechanisms of harm on biological systems from SARS-CoV-2 spike protein and vaccine-encoded spike protein for the purpose of exploring causes of COVID-19-associated harm, COVID-19 vaccine injury syndromes, and potential therapeutic strategies.

## Review

Methods

Article Layout

First, we categorized the findings of spike protein’s deleterious effects based on the organ system targeted: cardiovascular, hematological, neurological, respiratory, gastrointestinal, and immunological systems. Additionally, we detailed the differences between viral spike protein and vaccine-generated spike protein. Lastly, we explored potential detoxification strategies, including the McCullough protocol: base spike detoxification.

Search Strategy

We performed a literature search using the PubMed and Google Scholar databases. We also searched the grey literature, which is research produced by organizations that are not affiliated with traditional commercial or academic publishing and distribution channels. The primary search terms were "SARS-CoV-2 spike protein", "spike protein effects", "COVID-19 vaccine spike protein", "cardiovascular", “hematological”, “neurological”, “respiratory”, “gastrointestinal”, “immunological”, "detoxification", “treatment”, “degradation”, “curcumin”, “bromelain”, and “nattokinase”. These terms were used in varying combinations with filters applied for English language articles. All references cited in selected articles were manually searched to identify any additional relevant studies.

Study Selection and Eligibility Criteria

For investigating the harmful effects of spike protein, articles that specifically assessed the direct or indirect impacts of the spike protein on biological systems, in isolation or in relation to the SARS-CoV-2 virus or COVID-19 vaccination, were considered. We also included papers that helped assess the difference between viral spike protein and vaccine-generated spike protein. Lastly, we sought to include any published treatment protocols or compounds that had signals of benefit and acceptable safety profiles that may assist in spike protein detoxification. Original articles, in vitro studies, animal studies, and clinical studies published were included. Case reports, editorials, letters to the editor, and reviews were excluded unless they provided original results or synthesized available evidence.

Results

Deleterious Effects of Spike Protein by Organ System

**Figure 1:**
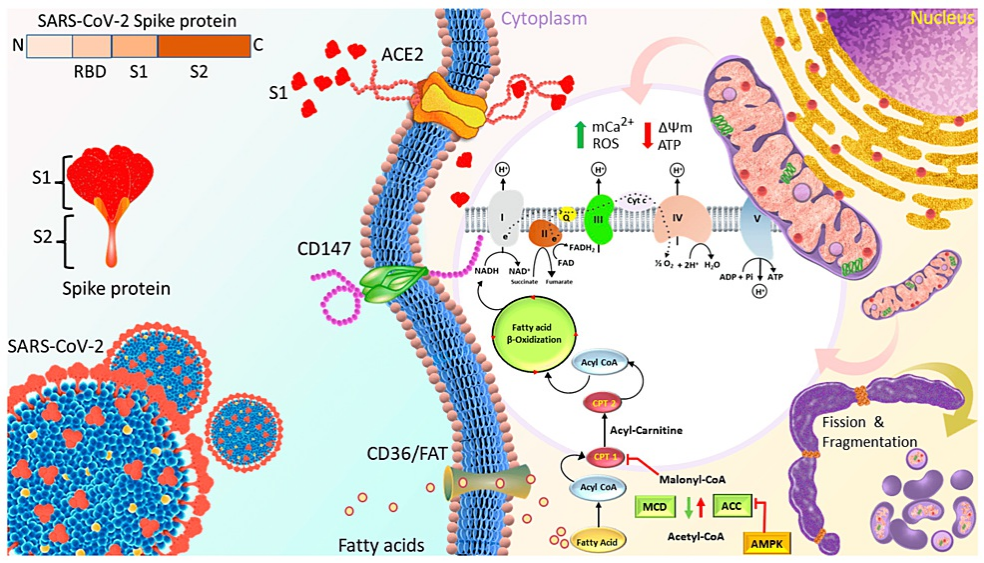
Proposed Mechanisms Through Which S1 of Spike Protein Induced Cardiac Mitochondrial Dysfunction, Which Leads to Cardiac Injury in COVID-19 Patients. Spike protein is the glycosylated protein that covers the surface of SARS-CoV-2 and binds to the host ACE2 receptor to mediate the viral cell entry. It is composed of S1 and S2 subunits that are responsible for ACE2 binding and membrane fusion, respectively. S1 possibly binds to ACE2 on the AC16 membrane and is then internalized into the cytosol and localized in organelles, such as mitochondria, which induces the transient increase in fatty acids transport and uptake for biogenetics, Δψm, and permanent mCa2+, and disrupts Δψm later, finally impairing mitochondrial function and promoting ROS production. In turn, ROS further exacerbates mitochondrial function and mitochondrial fragmentation. Moreover, S1 causes downregulation of TOM20; this effect might inhibit the pathways leading to mitochondrial biogenesis. Abbreviations: ACE2 = angiotensin-converting enzyme 2, FAT = fatty acid translocase, PCT1/2 = Carnitine palmitoyltransferase ½, MCD = Malonyl-CoA decarboxylase, ACC = acetyl-CoA carboxylase, AMPK = AMP-activated protein kinase, mCa2+ = mitochondrial calcium, Δψm = mitochondrial membrane potential, ROS = reactive oxygen species. *Figure and legend reprinted from Huynh et al. [14]. The legend title has been slightly adapted. Permission to use this figure has been granted in accordance with the open access Creative Common CC BY 4.0 license.

Cardiovascular system: The cardiovascular system has been identified as one of the primary targets of the SARS-CoV-2 spike protein. Disruptions in human cardiac pericytes, a type of cell that wraps around the endothelial cells of capillaries and venules, have been observed by the exposure of the spike protein. These disruptions include increased migration, secretion of inflammatory molecules that are seen in the cytokine storm, and creation of apoptotic factors that cause cell death [13]. Furthermore, spike protein has been found to hinder mitochondrial functions in human cardiomyocytes, potentially leading to energy deficits and reduced cardiac output. This effect may be due to spike protein inducing negative mitochondrial membrane potential, increasing intracellular calcium, and increasing levels of reactive oxygen species (ROS). Figure 1 illustrates this process [14]. The spike protein may also induce cardiomyocyte fusion, which may amplify the risk of arrhythmias, and irregular heart rhythms that can be life-threatening. In this study, spike protein induced delayed depolarizations, altered beating frequency, and induced calcium influx problems [15]. In the context of vaccination, there have been reports of spike protein's detection in patients diagnosed with COVID-19 vaccine-induced myocarditis [16,17]. From the available evidence, spike protein displays obvious toxic effects on the cardiovascular system.

**Figure 2:**
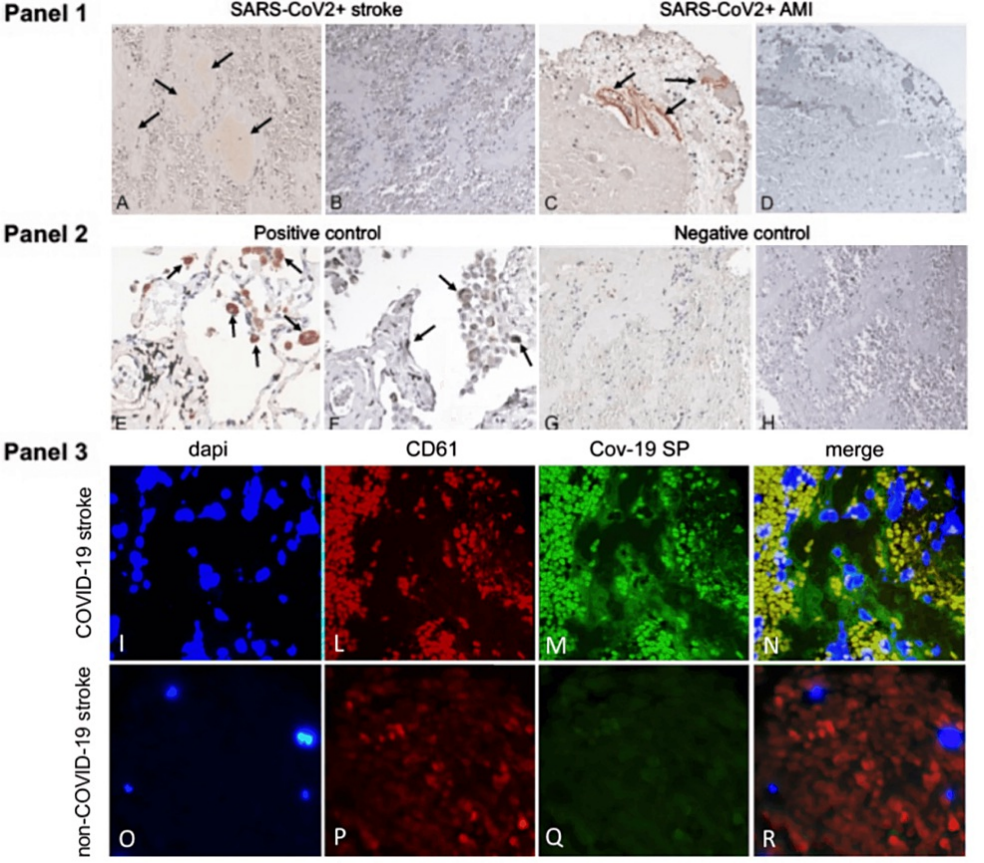
Arterial Thrombi From COVID-19 + Patients Contain SARS-CoV-2 Spike Protein But Not Nucleocapsid Protein. Panel 1. Immunostaining positive for SARS-CoV-2 spike protein (SP) (arrows) in representative thrombotic material from COVID-19 + patients, retrieved from cerebral (A) and coronary (C) arteries. Immunohistochemistry for nucleocapsid protein (NP) was negative in the same samples (B-D). Panel 2. Representative immunohistochemical staining positive for SP (E) and NP (F) (arrows) in the lung of a patient affected by COVID-19 (positive control). Representative immunostaining negative for SP (G) and NP (H) in a thrombus retrieved from the middle cerebral artery of a patient not affected by COVID-19 (negative control). Original magnification 20X. Panel 3. Double immunofluorescence of thrombotic material retrieved from COVID-19 and non-COVID-19 patients’ cerebral arteries. In the COVID-19 thrombus, platelets are co-stained with anti-CD61 (red-L,P) and anti-SARS-CoV-2 spike protein (SP) antibodies (green-M,Q), emitting yellow signals in the merged panel (N), while in the control (non-COVID thrombus), only the red CD61 signal is observed (R). *Figure and legend reprinted from De Michele et al. [21]. The legend title has been slightly adapted. Permission to use this figure has been granted in accordance with the open access Creative Common CC BY 4.0 license.

Hematological system: The hematological system, which includes blood and its components, is also targeted by the spike protein. The protein has been shown to incite inflammation in endothelial cells through a cascade involving integrin α5β1 and the NF-κB signaling pathway. This effect was also seen in vivo by intravenous inoculation of spike protein, which resulted in an increased expression of leukocyte adhesion molecules, coagulation factors, and proinflammatory cytokines in the lung, liver, kidney, and eye [18]. This inflammation can promote clot formation and hyperpermeability of the endothelial cell monolayer. Adding to the toxic effects, spike protein may transform fibrin into a form that resists fibrinolysis, making it harder for the body to dissolve blood clots. To do this, spike protein may induce structural changes to fibrin, complement 3, and prothrombin proteins [19]. These dangerous alterations can help explain the mechanism behind microclotting seen in COVID-19 patients. The spike protein's affinity to bind competitively to heparan sulfate, an important glycoprotein involved in coagulation, further aggravates coagulation and thrombosis pathologies [20]. In this study, researchers injected a zebrafish model with spike protein at a similar level as in a critically ill COVID-19 patient. They found that this directly induced blood coagulation and thrombosis [20]. Perhaps most concerning is the finding that free spike protein, in the absence of SARS-CoV-2 RNA and nucleocapsid, was found within blood clots in patients diagnosed with acute ischemic stroke (Figure 2) [21]. As with the cardiovascular system, spike protein exhibits a wide array of harmful effects on the hematological system.

Neurological system: Neurological implications of the spike protein are particularly concerning. Spike protein has been demonstrated to compromise the blood-brain barrier's integrity through RhoA activation, which can potentially allow harmful substances from the bloodstream to enter the brain [22]. This disruption is possibly achieved by upregulated ACE2 expression. Animal studies have revealed cognitive deficits and anxiety-like behaviors in mice following exposure to the spike protein, possibly due to non-cell autonomous death of neurons in the hippocampus [23]. Cell death of neurons was not directly caused by exposure to spike protein, but rather by spike protein’s activation of glial cells, leading to neurotoxicity [23]. On a cellular level, there are indications of spike protein possibly causing deleterious changes in molecular delivery and metabolic functions in brain endothelial cells [24]. In this paper, spike protein induced mitochondrial damage in brain cells, as also seen in cardiomyocytes [14,24]. Some experimental models even suggest that spike protein might be a contributing factor in long-term cognitive dysfunctions that mimic post-COVID-19 syndromes [25]. Corroborating the study mentioned earlier [23], Fontes-Dantas et al. found that brain infusion of spike protein induced neuroinflammation and hippocampal microgliosis, or over-activation of glial cells, by engulfment of synapses [25]. These data indicate that spike protein exerts potent harmful effects on the neurological system by damaging blood-brain barrier function and destroying neurons.

Respiratory system: Spike protein also targets the respiratory system. Spike protein has been shown to stimulate inflammatory pathways in human lung cells, specifically through MAPK and NF-kB activation [26]. This inflammatory cascade occurred independent of the virus, indicating that spike protein may have been the sole cause. Spike protein's influence extends to lung macrophages, where it triggers their activation [27]. The activation of lung macrophages was coupled with the promotion of efficient phagocytosis and dysfunction of intracellular calcium concentrations [27]. Such activations can exacerbate inflammatory responses in the lungs and may cause lung injury. Moreover, spike protein can induce dysfunctions in lung endothelial cells, leading to thrombo-inflammation, largely contingent on the C3a/C3a receptor signaling [28]. These dysfunctions include loss of thrombomodulin, von Willebrand factor increase, vascular and epithelial C3 deposits, and increased C3a receptor expression [28]. Spike protein is likely detrimental to the respiratory system, which is not surprising given that SARS-CoV-2 is a respiratory virus.

Gastrointestinal system: The gastrointestinal tract is not immune to spike protein's effects either. The protein can activate inflammatory signaling and cytokine production in intestinal epithelial cells [26]. This may result in gastrointestinal injury and digestive problems. Further, spike protein has been implicated in provoking intestinal inflammation by promoting VEGF production in enterocytes [29]. VEGF production was increased by activating Ras-Raf-MEK-ERK signaling and inducing intestinal permeability [29]. By triggering increased permeability and inflammation, spike protein may lead to leakage of toxins into circulation, resulting in negative systemic effects. Moreover, there has been a clinical case where the spike protein was identified in the intestinal cells of a patient diagnosed with COVID-19 multisystem inflammatory syndrome [30]. This finding provides evidence that spike protein may play a vital role in inducing inflammation in COVID-19 patients, particularly in the gut.

Immunological system: SARS-CoV-2 spike protein also exerts damaging effects on the immune system, particularly in monocytes, which are part of the innate immune system and are a type of white blood cell that helps destroy infected cells and regulate cellular homeostasis [31]. Recombinant spike protein induces significant monocyte structural abnormalities, including cytoplasmic intrusions, aberrant nuclei, dysmorphia, granulation, vacuolization, and platelet phagocytosis [32]. The deleterious effects were more severe with Alpha and Delta variant SARS-CoV-2 recombinant spike proteins compared to Omicron, indicating a correlation with clinical disease severity [32]. Moreover, spike protein may cause cytokine release syndrome, a hyper-inflammatory response, by activating human monocytes to produce cytokines [33]. This may lead to inflammatory conditions that cause multiple organ dysfunction. Ait-Belkacam et al. found that spike protein induced differing effects on monocytes between children and adults [34]. In this study, the authors concluded that spike protein may cause lower monocyte and B cell activation, along with higher inflammation, in adults compared to children [34]. Concerningly, spike protein can persist in monocytes for an indefinite period of time. In patients with post-acute sequelae SARS-CoV-2 infection (PASC or long COVID) and previous severe COVID-19 infection, spike protein was found in CD16+ monocytes for at least 15 months after initial infection [35]. The researchers used mass spectrometry techniques to confirm the presence of spike protein. Finally, spike protein was found to damage hematopoietic stem and progenitor cells (HSPCs) through hyperactivation of Nlrp3 inflammasome, resulting in pyroptosis, or cell death triggered by inflammation [36]. From the available data, it can be concluded that SARS-CoV-2 spike protein is detrimental to the immunological system by possibly causing long-term immune dysfunction.

Differentiating the SARS-CoV-2 Spike Protein From Vaccine-Generated Spike Protein

Vaccine-encoded spike protein differs from the viral version by the replacement of the amino acids lysine and valine with two or six proline amino acids, which stabilizes the spike conformation in an inactive prefusion state [37,38]. The purpose of this stabilization is that it makes the spike protein more immunogenic than the viral version, thus making it more effective at inducing antibody production. Even though stabilized prefusion spike protein is effective at invoking an immune response, it may not be safer than its viral counterpart. Spike protein from the virus is cleaved at the S1/S2 boundary, thus causing smaller fragments. mRNA vaccines encode for the full-length prefusion protein that is not cleaved [4]. These structural differences may contribute to spike protein pathogenicity. Yonker et al. [16] showed that a vaccine-generated spike was found unbound by antibodies in those with post-vaccine myocarditis and not in controls. Furthermore, Baumeier et al. [17] found stabilized prefusion spike protein in cardiomyocytes via biopsy in patients with vaccine-induced myocarditis while ruling out infectious causes. Brogna et al. [39] found the stabilized prefusion spike protein in human subjects for up to six months after vaccination using mass spectrometry techniques. This extensive persistence of vaccine-generated spike protein in humans is also seen with COVID-19 infection spike protein, as shown by Patterson et al.’s [35] and Craddock et al.’s [40] findings that spike protein can be found in human subjects for at least a year after acute infection in those with post-acute sequelae. Perry et al. [12] concluded that spike protein is pathogenic from both SARS-CoV-2 and vaccine mRNA. These data indicate that the stabilized prefusion spike proteins can persist in the human body, be difficult to break down by natural human mechanisms, and may possess similar harmful characteristics to the viral type.

Rationale for SARS-CoV-2 and COVID-19 Vaccine Spike Protein Detoxification

Given the deleterious effects of spike protein [13-36] and its persistence [35,39,40] in those with post-acute sequelae after SARS-CoV-2 infection and COVID-19 vaccination, strategies to eliminate spike protein from the human body may be warranted to reduce the burden of disease from COVID-19 and vaccine injury syndromes. As revealed by autopsy, some severe COVID-19 vaccine injury syndromes that may be directly or indirectly caused by spike protein include sudden cardiac death, myocardial infarction, myocarditis, pericarditis, pulmonary embolism, vaccine-induced immune thrombotic thrombocytopenia, brain hemorrhage, multi-organ failure, respiratory failure, and cytokine storm [12,41]. Some complications from the SARS-CoV-2 virus and COVID-19 that may be caused by spike protein include myocardial infarction, pulmonary embolism, deep vein thrombosis, aortic thrombosis, acute limb ischemia, and disseminated intravascular coagulation [42]. As mentioned earlier, spike protein was found residing in heart tissue in patients with post-vaccination myocarditis [17] and in blood clots in patients with acute ischemic stroke [21]. Most of the injury syndromes involve cardiac and thrombotic events that have been associated with spike protein, revealing it as a prime target for treatment. At the time of writing, specific and widely established treatment protocols to remove spike protein from the human body are non-existent. Thus, it is essential to conduct further investigation and create effective treatment strategies for the management of spike protein-related pathologies.

McCullough Protocol: Base Spike Detoxification

**Figure 3:**
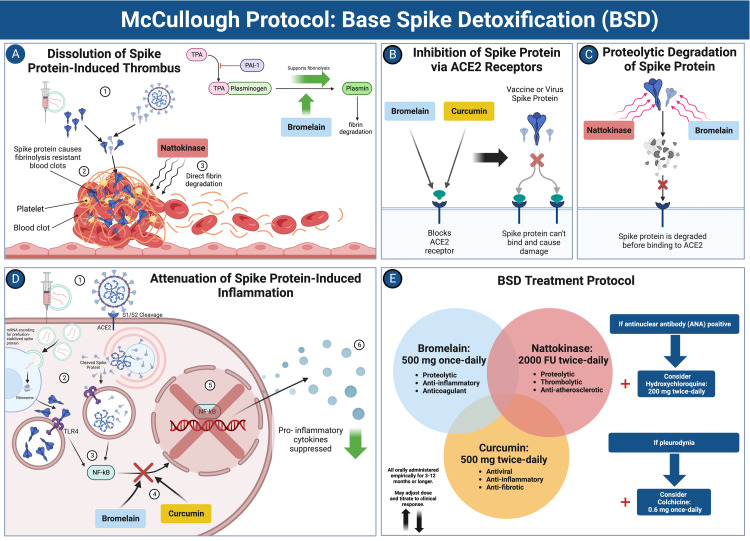
McCullough Protocol: Base Spike Detoxification (BSD). A: Dissolution of spike protein-induced thrombus. Nattokinase directly degrades fibrinolysis-resistant fibrin (from spike protein), and bromelain upregulates fibrinolysis. B: Inhibition of spike protein via ACE2 receptors. Bromelain and curcumin block the ACE2 receptor, preventing spike protein from binding. C: Proteolytic degradation of spike protein. Nattokinase and bromelain degrade spike proteins, rendering them inactive. D: Attenuation of spike protein-induced inflammation. Bromelain and curcumin downregulate the NF-kB signaling pathway induced by spike protein, leading to the suppression of inflammatory molecules. E: BSD treatment protocol. The full treatment regimen and the addition of other compounds based on clinical indication are illustrated. Abbreviations: TPA = tissue plasminogen activator, PAI-1 = plasminogen activator inhibitor-1, ACE2 = angiotensin converting enzyme-2, NF-kB = nuclear factor kappa B, S1/S2 = spike protein subunits S1/S2, TLR4 = toll-like receptor 4. *Created with BioRender.com. Panel E adapted from McCullough et al. [43].

McCullough et al. recently published (2023) the first rationale for spike protein detoxification, called the McCullough protocol: base spike detoxification [43], that holds considerable promise. The protocol includes a natural triple-agent oral regimen of nattokinase, bromelain, and curcumin that provides four putative, primary mechanisms of action: 1) proteolytic degradation of spike protein, 2) inhibition of inflammation from spike protein and its fragments in tissues, 3) dissolution of microthrombi, and 4) anticoagulation. Figure 3 illustrates the base spike detoxification protocol and its mechanisms.

The full base spike detoxification protocol is as follows [43]:

Bromelain 500 mg once a day, nattokinase 2,000 FU twice a day, and curcumin 500 mg twice a day. The regimen is to be followed for 3-12 months or more, depending on disease resolution progress. These are initial dosages and may be adjusted in accordance with the tolerability and severity of injury syndrome. Because doses are far below known limits of safety, dose escalation would be reasonable if there are residual symptoms after three months of therapy. If ANA is positive and an autoimmune disease is suspected, prescribed hydroxychloroquine 200 mg twice a day should be added to the regimen. If pleurodynia or atypical chest pain is present, prescribed colchicine 0.6 mg once a day should be used in addition.

**Figure 4:**
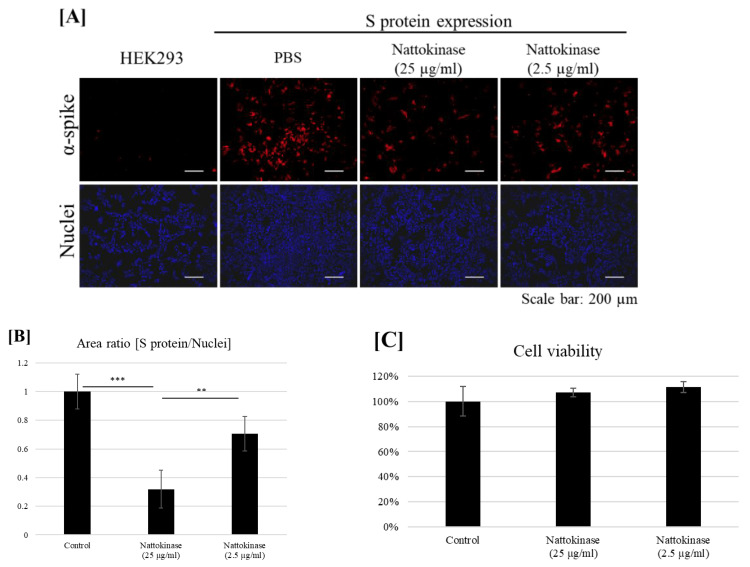
Degradative Effect of Nattokinase on Spike Protein. (A) Degradative effect of nattokinase on spike protein on the cell surface. Spike-pcDNA3.1 was transfected with HEK293 cells and incubated for 9 h. After incubation, nattokinase (25 and 2.5 µg/mL) was added to the culture medium and further incubated for 13 h. Cells were fixed, and immunofluorescent analysis was performed. S protein on the cell surface was stained with an anti-spike protein antibody (red), and the nucleus was stained with DAPI (blue). (B) Ratio of the S protein area to the nucleus positive area. Three images per sample were captured, and S protein/nucleus positive areas were calculated. Data are shown as mean + SD, and the p-value was determined by one-way analysis of variance (ANOVA) with Tukey’s post-hoc test using R software (R-3.3.3 for Windows) (** p < 0.01; *** p < 0.001). (C) Cell viability was evaluated by an MTT assay. Indicated nattokinase was added to the culture medium and incubated for 13 h; an MTT assay was performed. *Figure and legend reprinted from Tanikawa et al. [47]. The legend title has been slightly adapted. Permission to use this figure has been granted in accordance with the open access Creative Common CC BY 4.0 license.

*Nattokinase*, a proteolytic enzyme derived from the fermentation of soybeans by Bacillus subtilis natto [44], has been traditionally used in Japan for cardiovascular benefits and possesses direct fibrinolytic activity by hydrolyzing fibrin and plasmin substrate, meaning it can be used to dissolve blood clots [45]. This makes nattokinase a vital tool to dissolve spike protein-induced blood clots, as they may contain fibrin that is resistant to fibrinolysis [19]. Additionally, nattokinase has a potent degrading effect on the spike protein of SARS-CoV-2 [46,47]. Figure 4 shows the degradative effect of nattokinase on spike protein on the cell surface [47]. The extensive persistence of spike protein [35,39,40] indicates that external drugs, specifically protein-degrading enzymes such as nattokinase, may be needed to degrade it in the human body. Kurosawa et al. demonstrated that, in humans, D-dimer levels were notably increased at both six and eight hours, while blood fibrin/fibrinogen breakdown products showed a significant rise four hours post the intake of a one-time oral dose of 2,000 FU (100 mg) (p < 0.05) [48]. Based on these findings, a suggested initial dosage might be 2,000 FU administered twice daily. Nattokinase has been shown to be largely safe other than possible excessive bleeding especially when combined with other medications [48].

*Bromelain*, a proteolytic enzyme sourced from the stem of pineapples [49], has been traditionally hailed for its healing and anti-inflammatory capabilities, particularly in cases of arthritis and injury. Of significance is bromelain's anticoagulant activity. It downregulates PGE-2 and thromboxane A2, promoting a relative prostacyclin abundance in platelets. Furthermore, it aids in fibrinolysis by promoting plasminogen conversion to plasmin and inhibiting platelet aggregation [50]. Kritis et al. demonstrated that bromelain can obstruct SARS-CoV-2's entry into cells by cleaving its spike protein and reducing ACE2 and TMPRSS2 expression [51]. This enzyme can also hydrolyze glycosidic linkages, which comprise spike protein’s glycosidic shield that helps protect it from immune responses [52]. To attenuate inflammation, bromelain, in part, downregulates the pro-inflammatory prostaglandin E−2 (PGE-2) through inhibition of NF-kB and cyclooxygenase 2 and inhibits inflammatory mediators [51]. Thus, bromelain exerts multiple mechanisms of action against spike protein’s toxic effects and persistence. Bromelain has been used as a daily dosage of 200-2,000 mg; thus, 500 mg is a suggested initial dose [53]. Bromelain is mainly safe with low toxicity, but it can amplify bleeding risk and affect the absorption rate of several medications, potentially leading to drug interactions [54].

*Curcumin*, a polyphenol extracted from turmeric, is renowned for its anti-inflammatory properties and its ability to modulate inflammation during viral infections. Curcumin also supports fibrinolysis and the process of anticoagulation [51]. Beyond its traditionally recognized benefits, curcumin has shown promising antiviral actions against a wide range of viruses, including influenza, hepatitis, and notably, SARS-CoV-2 [55]. It achieves this by obstructing the spike protein's binding sites (ACE2 receptors and TMPRSS-2). Curcumin’s anti-inflammatory effects are realized through inhibiting NF-κB signaling [56]. An in-silico study found that curcumin can inhibit the spike protein of the Omicron variant through interaction with its amino acids [57]. Randomized trials have consistently indicated decreases in high-sensitivity C-reactive protein (hs-CRP) and other markers of inflammation in situations involving spike protein-induced infections or injuries [58,59]. Curcumin is non-toxic at doses up to 8,000 mg a day [60]. Large doses, particularly with ill-absorbed formulations, can lead to gastric complications [61]. Enhanced absorption of curcumin is achieved in combination with piperine, or with nano or liposomal formulations, which are available as over-the-counter oral supplements. Doses vary widely depending on the formulation, but 500 mg twice a day has been shown to be a common and safe dosage regardless of curcumin type [61].

*Hydroxychloroquine*, a well-known FDA-approved antimalarial and anti-inflammatory, adds additional support for immunocompromised patients by inhibiting the binding of spike protein to human cells [62]. A real-time meta-analysis of 413 published peer-reviewed studies for hydroxychloroquine as a treatment for COVID-19, including a total of 529,687 patients, shows a statistically significant lower risk for mortality and hospitalization, along with accelerated viral clearance [63]. This effect was the strongest when patients were treated early, indicating the importance of early treatment. Since hydroxychloroquine accelerates viral clearance, it subsequently assists in spike protein removal, and it may be a great addition to base spike detoxification. This compound has been found to be well-tolerated, safe, and not associated with a risk of ventricular arrhythmia at a dose of 200 mg twice a day provided that the expected prolongation of QTc is managed along with other drugs with serial ECGs. Gastrointestinal symptoms may occur [64].

*Colchicine*, an FDA-approved alkaloid found in the plants Colchicum autumnale and Gloriosa superba, has been traditionally used in therapeutics for its anti-inflammatory properties [65]. This compound can reduce the risk of myocardial infarction and stroke [66]. Moreover, colchicine may reduce myocardial injury in the presence of spike protein [67]. Pleurodynia has been diagnosed post-COVID-19 vaccination and may be indicative of cardiac inflammation [68]. The COLCORONA trial demonstrated that colchicine was safe and had a favorable impact on COVID-19 and its immediate post-acute sequelae. In patients with PCR-confirmed COVID-19, colchicine lowered the rate of hospitalization and death compared to placebo [69]. A meta-analysis of five randomized trials, including a total of 16,048 patients, found that colchicine decreased COVID-19 severity and decreased C-reactive protein (CRP), indicating its potent anti-inflammatory effect in the presence of spike protein [70]. Thus, the addition of colchicine is indicated when a patient presents with pleurodynia post-COVID-19 vaccination or post-infection. Moreover, 0.5 mg twice daily has been shown to be a safe and effective dosage for the treatment of COVID-19 [65,69,70]. 

Additional compounds that may assist in spike protein detoxification and degradation include the following:

N-Acetylcysteine (NAC): It dissolves spike protein through the destruction of disulfide bonds and prevents binding at ACE2 [52,71,72].

Glutathione: It disrupts spike protein disulfide bonds [72].

Ivermectin: It binds and inhibits spike protein [73].

Quercetin: It binds and inhibits spike protein [74].

Apigenin: It binds and inhibits spike protein [74].

Nicotine: It disrupts glycosylation on spike protein and blocks possible spike protein-nicotinic cholinergic receptor interaction [75,76].

Emodin: It blocks the spike protein-ACE2 interaction [77].

Fisetin: It binds and inhibits spike protein [78].

Rutin: It binds and inhibits spike protein [79].

Silymarin: It binds and inhibits spike protein [80].

Discussion

We found abundant evidence that SARS-CoV-2 spike protein may cause biological damage in the cardiovascular, hematological, neurological, respiratory, gastrointestinal, and immunological systems [13-36]. Mechanistically, spike protein has been shown to cause dysfunction in many cell types by causing metabolic deteriorations, leading to cell death. Stabilized prefusion (vaccine-derived) spike protein may possess similar harmful mechanisms as viral spike protein. Spike protein is found, in the absence of nucleocapsid, directly in cardiomyocytes and blood clots in patients diagnosed with myocarditis and acute stroke, respectively [17,21]. Vaccine-derived and viral spike proteins have been found in humans for at least 6-15 months after vaccination or infection [35,39,40] in those with post-acute sequelae, indicating spike protein as a possible primary contributing factor to long COVID, with substantial persistence in human systems due to the absence of an innate clearing mechanism. However, more research is needed to further investigate the effect of spike protein in patients with post-acute sequelae after COVID-19 and COVID-19 vaccination. Meanwhile, the current data points to a strong signal to urgently develop spike protein detoxification protocols.

Although methods to degrade or block spike protein have been investigated [46,47,51,52,56,57,62,71-80], there are currently no widely accepted protocols to do this in human subjects. The McCullough protocol: base spike detoxification is the first protocol established to help remove spike protein derived from SARS-CoV-2 infection and vaccination in humans. The three-drug regimen of nattokinase, bromelain, and curcumin was chosen due to their proven safety records, as well as their anti-inflammatory and anti-coagulant properties combined with their synergistic and potent effects in degrading and inhibiting spike protein [44-61]. This protocol may be useful in the attenuation of COVID-19 vaccine-induced injury syndromes and long-term COVID-19 complications.

The addition of other agents that can further assist in the detoxification of spike protein [62-80] may be indicated based on clinical outcomes. Most notably, the addition of N-acetylcysteine (NAC) may add an extra mechanism of action against spike protein to the protocol. NAC has been shown to disrupt disulfide bonds of the spike protein, rendering it unable to bind to the ACE2 receptor [71,72]. Moreover, the combination of NAC with bromelain has been shown to synergistically disrupt spike protein by breaking glycosidic linkages and disulfide bonds [52]. NAC has a well-established safety profile and is commonly taken at a dose of 600 mg twice a day [81]. Thus, the addition of NAC to the regimen of bromelain, curcumin, and nattokinase may be useful for individuals with severe treatment-resistant COVID-19 or COVID-19 vaccine-related complications. Since all of these compounds can reduce blood clotting, patients should be counseled and monitored for bleeding complications, including easy bruising, nasal mucosal bleeding, and gastrointestinal hemorrhage. Self-administration is not advised without physician oversight.

The base spike detoxification protocol was devised based on the best evidence currently available. No therapeutic claims can be made until large-scale, prospective, randomized, double-blind, placebo-controlled trials are completed. On November 15, 2023, we searched clinicaltrials.gov and found no planned or ongoing trials with nattokinase or bromelain in the treatment of post-acute sequelae after COVID-19 or vaccination. However, there is a planned trial to assess the effect of curcumin, in conjunction with boswellia and vitamin C, on long COVID [82]. Thus, base spike detoxification is an important advancement in the development of testable hypotheses for future trials assessing treatments for SARS-CoV-2 infection and post-COVID-19 vaccination injury syndromes.

## Conclusions

SARS-CoV-2 spike protein is a highly persistent, potentially pathogenic substance that may incite inflammation and tissue damage in almost all organ systems, resulting in post-acute sequelae. The vaccine-generated spike protein is different from the viral type, but both have been associated with deleterious effects and persistence in biological systems. Thus, therapeutics that target spike protein may be essential in treating COVID-19, its long-term effects, and possibly COVID-19 vaccine injury syndromes. Base spike detoxification is a promising proposal designed to theoretically attenuate spike protein and its associated damage. However, more pre-clinical and clinical research is needed culminating with large-scale, prospective, randomized, double-blind, placebo-controlled randomized trials to fully assess safety and efficacy. Moreover, further investigation is essential to ensure vaccine-produced, stabilized prefusion spike protein safety and half-life in humans and that it does not possess the same deleterious effects as the viral spike protein.
